# The Availability, Pricing, and Affordability of Essential Diabetes Medicines in 17 Low-, Middle-, and High-Income Countries

**DOI:** 10.3389/fphar.2019.01375

**Published:** 2019-11-19

**Authors:** Zaheer-Ud-Din Babar, Sara Ramzan, Faris El-Dahiyat, Ilias Tachmazidis, Adeola Adebisi, Syed Shahzad Hasan

**Affiliations:** ^1^Department of Pharmacy, University of Huddersfield, Huddersfield, United Kingdom; ^2^College of Pharmacy, Al Ain University of Science and Technology, Al Ain, United Arab Emirates; ^3^Department of Computer Science, University of Huddersfield, Huddersfield, United Kingdom

**Keywords:** affordability, availability, diabetes, essential medicines, primary care pharmacy

## Abstract

**Background:** One third of the world population does not have access to essential medicines. Diabetes require a long-term therapy, which incurs significant health care cost and thus impact access and affordability. This study aims to assess the availability, prices, and affordability of four essential medicines used to treat diabetes in private primary care pharmacies in 17 countries.

**Methods:** Data on affordability, availability, and prices of four essential diabetes medicines from 51 primary care pharmacies across 17 countries were obtained using a variation of the World Health Organization/Health Action International (WHO/HAI) methodology. The surveyed countries were Oman, Qatar, Saudi Arabia, United Arab Emirates, China, Jordan, Russia, Armenia, Bangladesh, Egypt, Georgia, India, Pakistan, Sri Lanka, Afghanistan, Nepal, and Tanzania. International reference prices and daily income of the lowest-paid unskilled government workers were used as comparators. The prices were converted into US$ using both foreign exchange rates and purchasing power parity. We compared patterns of affordability and availability and prices of innovator brand (IB) and lowest priced generic (LPG) of diabetes medicines by WHO regional groupings and by country level.

**Results:** Lowest priced generic of metformin 500 mg had the highest total mean availability (≥80%) among all the surveyed medicines. The total mean availability of insulin 100 IU/ml was only 36.21% (IBs and LPGs), where IB was more frequently available than LPG (50% *vs*. 26%) across 17 surveyed countries. Patients would have to spend more to procure 1-month’s supply of IB of insulin in low-income than patients in high-income countries (no. of day’s wages: 2.37 *vs*. 0.46, p = 0.038). For the majority of the surveyed countries the median price-ratio was less than 3. The highest PPP-adjusted prices for 30-day treatment with IB of insulin 100 IU/ml and metformin 500 mg were highest in Bangladesh ($80.21) and Tanzania ($4334.17), respectively.

**Conclusion:** Availability of generic form of insulin is poor; IB of insulin was more affordable in high-income countries than low-income countries. Most of the LPGs was reasonably priced and affordable to the lowest-paid unskilled worker.

## Introduction

The global prevalence of diabetes mellitus among adults over 18 years of age is expected to increase from 4.4% in 1980 to 8.8% in 2017 (Mathers and Loncar, 2006; IDF, 2018). Type 2 diabetes mellitus (T2DM) represents 90% of diagnosed cases of diabetes and represents a potential global epidemic (IDF, 2018). The prevalence of T2DM is increasing and is likely to affect 500 million people worldwide by 2030 with up to 1 in 8 adults suffering from the disease (IDF, 2018). Affordability and availability of medicines are growing challenges for healthcare systems all over the world (WHO, 2018a). In developing countries, the expenditure on medicines represents 25–66% of the total public and private health spending, and hence accounts for the biggest household expense after food (WHO, 2018b).

Globally, spending on diabetes medicines by healthcare systems and individuals has increased and this represents a significant proportion of healthcare budgets (WHO, 2018a). The new insulin analogues and oral diabetes medicines have been associated with high expenditures, both for individuals and governments (Beran et al., 2018) and the cost of these medicines are considered a major challenge in accessing these medicines particularly in low- and middle-income countries (LMICs) (McEwen et al., 2017).

For the past 40 years, the World Health Organization (WHO) has been at the forefront of promoting equality in access to medicines in high-income countries and LMICs. Based on efficacy, safety, and affordability of medicines the WHO has developed an essential medicine list (EML). The medicines on this list are defined as: “*…those that satisfy the priority health care needs of the population*” (WHO, 2018b). Some high-income countries have also introduced mechanisms to improve medicines access such as wise list in Stockholm, Sweden, with initially just 200 plus medicines for ambulatory care (now expanded to include hospital outpatients) developed on the premise that most physicians only know 200 to 300 medicines well (Gustafsson et al., 2011). As a specialized agency concerned with international public health, World Health Organization/Health Action International (WHO/HAI) have been working in the last decade or so to collect evidence to measure and promote affordability and availability of medicines, particularly in LMICs (Gelders et al., 2006; Mendis et al., 2007; Cameron et al., 2009; van Mourik et al., 2010; Mori and Robberstad, 2012; Dabare et al., 2014; Mori et al., 2014; Wang et al., 2017; Babar et al., 2013).

Recent research suggests that a month’s treatment with oral diabetes medicines is available at low cost in middle- and high-income countries (Cameron et al., 2009; Dabare et al., 2014; Mendis et al., 2007). Across LMICs, a growing body of literature has evaluated the availability and prices of diabetes medicines (Babar et al., 2007; Cameron et al., 2009; Dabare et al., 2014; Wang et al., 2017; WHO, 2018c) but the prices reported in these studies may not reflect what patients and governments are actually paying for these medicines. However, some recent studies did explore the availability and affordability of essential medicines to treat non-communicable diseases (Ewen et al., 2017; Wang et al., 2017; Beran et al., 2018).

Unaffordable medicine prices and poor availability are among many factors that affect access to medicines (Opinions of the Communist Party of China Central Committee and the State Council on Deepening the Health Care System Reform, 2009). Low availability and low affordability of medicines have the potential to affect a significant proportion of the world population. Low availability of medicines can be due to several factors such as high prices, poor prescribing practices of both innovator brands and generics, ineffective supply systems, and lack of patient compliance (Mendis et al., 2007). Evidence shows that about half of the population in the poorest parts (such as in China) cannot afford essential medicines, mainly due to the high or unaffordable costs (Opinions of the Communist Party of China Central Committee and the State Council on Deepening the Health Care System Reform, 2009). More than 95% of the drugs on WHO EML are no longer patented however it is estimated that a large number of people still do not have regular access to medicines (World Health Organization, 2004; Ewen et al., 2017; Beran et al., 2018). This means a gap in the access to medicine exists. It is also important to emphasize that a low procurement price does not always translate into a low cost to the patient.

In this context, the aim of this study was to measure availability, pricing, and affordability of four essential diabetes medicines in private primary care pharmacies in 17 low-, middle, and high-income countries. Measuring the prices and availability of medicines will contribute toward an enhanced understanding of actual prices patient pays at private sector retail pharmacies, and affordability of essential medicines for diabetes treatment. Informed prices would allow government and funding agencies to negotiate prices with pharmaceutical companies (World Health Organization, 2004; Cameron et al., 2009).

## Methods

### Sampling Strategy

A survey was conducted to examine the availability, pricing, and affordability of selected essential diabetes medicines. Low- and middle-income countries were the target countries for this study. A purposive sampling method was used to collect data (HAI, 2008). The surveyed countries were categorized into four groups. The World Bank income group 2018 was used to define the income status of each of the chosen countries (The World Bank, 2018a; The World Bank, 2018b; The World Bank, 2018c; The World Bank, 2018d).

### Data Collection and Survey Tool

A data collection form was adapted from WHO/HAI standard methods measuring the price and availability of selected diabetes medicines (World Health Organization, 2004; HAI, 2008). The developed data collection form had three sub sections; the first section was on demographics of the country, the second section was on the medicine outlet, and last section was on medicine prices and availability of four medicines where the international reference prices (IRP) were available; metformin 500 mg, metformin 850 XR, gliclazide 80 mg, and insulin 100 IU/ml. These products were selected based on our consultation with the collaborators in sampled countries.

Data were collected between March and June 2018. The project described in an invitation letter was emailed to selected individuals and groups (academics, pharmacists, and other healthcare professionals). In case no response was received following the initial email, a follow-up email was sent 2 weeks later. When written consent for participation was received, another email was sent with the data collection tool and supplementary information including data collection information sheets, and a final checklist. The “checklist” specified that by providing the requested data consent was deemed to be given by the data collector. Data collectors were provided written instructions on how to collect data. All data collectors were given the option to contact a team member to go through the data collection process.

Data were collected by making one visit to at least two private primary care pharmacies in the capital or main city in each participating country. Each data collector was instructed to collect pharmacy level prices for each of the four selected medicines for innovator brand (IB) and lowest priced generic (LPG) (**Table 1**). The price and availability of each medicine was recorded using the provided data collection forms. Furthermore, the national currency, exchange rate expressed in US dollars on the day of data collection along with daily wage of the lowest-paid unskilled government worker was recorded.

**Table 1:** Four essential medicines used to treat diabetes included in the survey.

Medicine name	Brand	Indication	Medicine strength	Dosage Form	WHO essential medicine list
Metformin	Glucophage	Diabetes	500 mg	Tablet or capsule	On the WHO core list in the same dosage form and strength On the WHO complementary list in same dosage form and strength
Metformin XR	Glucophage	Diabetes	850 mg XR	Tablet or capsule	On the WHO list in different dosage form or strength
Gliclazide	Diamicron	Diabetes	80 mg	Tablet or capsule	On the WHO core list in the same dosage form and strength
Insulin human soluble	Novo Nordisk	Diabetes	100 IU/ml	Injection	On the WHO core list in the same dosage form and strength

Upon receipt of data it was screened for completeness and accuracy. This was achieved by checking the brand names of the IB as manufacturers do not use the same trading names across countries. Brand names for the IB were checked and accepted as appropriate. Currency exchange and wages of the lowest-paid unskilled government workers were double checked by the authors. In cases where data was missing or unclear the data collector was contacted for clarification.

### Availability Assessment

In each surveyed facility the availability of IB and lowest priced generic were recorded for the selected medicines using Microsoft^®^ Excel. The availability was recorded as being i) available on survey date or ii) not available on survey date, regardless of the available pack size. Differences in availability between innovator and generic product of a given medicine on its own and the mean availability will determine if the availability differs significantly. The availability in each facility was described using the following range (Gelders et al., 2006): very low (≤30), low (Syhakhang, 2001; Kotwani, 2009; Shi et al., 2010; Babar et al., 2011; Toverud et al., 2015; Xi, 2015; Beran et al., 2016; Flood et al., 2017; Liu et al., 2017; Nordisk, 2017; WHO Collaborating Centre for Drug Statistics Methodology, 2018), fairly high (50–80), and high (≥80). For each medicine it was recorded if the medicine was listed on the WHO EML (**Table 1**).

### Price-Ratio Comparison

The WHO/HAI methods for comparison of local prices with supplier median unit price (USD) listed in Management Sciences for Health’s Drug Price Indicator Guide was adapted for this study (MSH, 2015). Where no median supplier prices were available, the listed buyer median unit prices were used. A median price ration (MPR) for each country was calculated using the lowest generic prices for each medicine. The IRP is an indicator for how costly the medicine is. If the calculated ratio is ≤1 the reported price is considered to be reasonable in public sector. A value ≥3 in the private sector means the local’s pay more for the medicine than recommended by WHO (World Health Organization, 2004; HAI, 2008).

### Affordability Assessment

The affordability of each medicine was calculated for each country separately using the price of the lowest priced medicine irrespective of brand. The estimate is based on the number of days wages, the lowest-paid unskilled government worker would require to purchase 30 days of treatment at a standard dose. The WHO recommends that for a treatment to be affordable it should not exceed 1 day’s wages using the wage of the lowest-paid unskilled government worker. It is important to note that the estimate is based on if the patient were to pay for the treatment out-of-pocket and thus other arrangements such as private insurance and government funding has not been considered in this estimate.

### Currency Conversion

The prices of IBs from local currencies were converted to US$ using both foreign exchange rates and purchasing power parity (PPP). In foreign exchange rates method, we used the foreign exchange rates (FOREX) on September 24^th^, 2018 to perform the conversion (Forex exchange rates). In PPP, we used PPP rates provided by the World Bank to perform the conversion (World economic outloook). Rates for PPP are estimated based on the cost of purchasing a similar basket of goods in different countries. Unlike FOREX, PPP values do not fluctuate significantly with time (Shi et al., 2010).

### Statistical Analysis

The data were analyzed using SPSS version 24 with 0.05 as level of significance. The price-ratio, availability, and affordability were presented as means and standard deviations. The differences in price-ratio and affordability between countries grouped into four categories based on their income level were calculated using Kruskal-Wallis test and Mann-Whitney for pairwise comparisons.

## Results

Out of 50 countries approached, 20 (40.0 percent) agreed to participate and provided the data in the study. The surveyed countries were Oman, Qatar, Saudi Arabia, United Arab Emirates, China, Jordan, Russia, Armenia, Bangladesh, Egypt, Georgia, India, Pakistan, Sri Lanka, Afghanistan, Nepal, and Tanzania. Of 20 countries, data collection forms from 3 countries were excluded due to inadequate data set.

### Surveyed Primary Care Pharmacies

Using a standardized approach, the availability of medicines in 51 primary care pharmacies across seventeen countries were surveyed. **Table 2** shows the countries where the survey samples were successfully collected along with their WHO region and income level.

**Table 2:** Countries included in the survey.

Country	WHO regions	Income level (The World Bank, 2018b; The World Bank, 2018c; The World Bank, 2018d; MSH, 2015)	Surveyed pharmacies per country
Afghanistan	Eastern Mediterranean	Low income	3
Armenia	Europe	Lower middle income	3
Bangladesh	South-East Asia	Lower middle income	3
China	Western Pacific	Upper middle income	3
Egypt	Eastern Mediterranean	Lower middle income	2
Georgia	Europe	Lower middle income	3
India	South-East Asia	Lower middle income	2
Jordan	Eastern Mediterranean	Upper middle income	3
Nepal	South-East Asia	Low income	3
Oman	Eastern Mediterranean	High income	3
Pakistan	Eastern Mediterranean	Lower middle income	3
Qatar	Eastern Mediterranean	High income	2
Russia	Europe	Upper middle income	3
Saudi Arabia	Eastern Mediterranean	High income	4
Sri Lanka	South-East Asia	Lower middle income	3
Tanzania	Africa	Low income	3
United Arab Emirates	Eastern Mediterranean	High income	3

### Availability of Medicines

The total mean availability of four medicines (IBs and LPGs) on the day of the survey was lowest in Georgia (29%) and highest in Pakistan (88%) as presented in **Table 3**. No IBs (of four surveyed medicines) were available in Bangladesh and Nepal. The total mean percentage availability of IBs was very low (≤30%) in Armenia, Egypt, India, and Tanzania. For LPGs, only two countries (Georgia and Saudi Arabia) had total mean percentage availability less than 30%.

**Table 3:** Median percentage availability of selected diabetes medicines in each country and mean percentage availability of innovator brand and lowest priced generic in each country

Medicines		Afghanistan	Armenia	Bangladesh	China	Egypt	Georgia	India	Jordan	Nepal	Oman	Pakistan	Qatar	Russia	Saudi Arabia	Sri Lanka	Tanzania	United Arab Emirates	Total mean percentage availability
Metformin 500 mg	IB	100	100	0	33	33	100	0	100	0	100	100	100	100	100	67	33	100	69
LPG	100	100	100	100	33	100	66	66	100	100	100	100	100	100	50	100	100	100	89
Metformin 850 mg XR	IB	67	0	0	0	0	33	0	0	0	33	0	0	100	50	33	0	0	19
LPG	33	33	0	100	33	100	33	67	0	100	66	100	33	100	0	33	100	0	53
Gliclazide 80 mg	IB	0	0	0	67	33	0	0	0	0	0	100	33	0	50	67	0	0	21
LPG	0	0	20	100	67	100	0	67	0	100	100	100	33	0	0	100	0	0	46
Insulin 100 IU/ml	IB	33	0	0	67	0	0	100	100	0	0	100	100	33	50	100	100	67	50
LPG	0	0	40	0	33	0	0	50	100	0	0	100	0	0	25	33	0	0	26
Total mean percentage availability		42	33	38	42	46	29	44	50	38	50	88	50	54	41	63	38	50	
Total mean percentage availability of IB		50	25	0	42	17	33	25	50	0	33	75	58	58	63	67	25	50	
Total mean percentage availability of LPG		33	40	75	42	75	25	63	50	75	67	100	42	50	19	58	50	50	

≤30, very low; 30–49, low; 50–80, fairly high; ≥80, high.

The total mean availability of insulin 100 IU/ml was 36.21% (IBs and LPGs), with the branded product (50% total mean availability) of insulin 100 IU/ml more frequently available than LPG (26% total mean availability) across 17 surveyed countries. LPG of insulin was not available in 10 out of 17 surveyed countries on the day of survey. Both IB and LPG of insulin were not available in Bangladesh, Egypt, Georgia, Nepal, Oman on the day of the survey. The total availability of IBs was particularly low for metformin 850 XR (19%) and gliclazide 80 mg (21%). The total availability of LPGs was also low for insulin 100 IU/ml (26%). There was a high mean availability of metformin 500 mg (89% of LPG *vs*. 69% of IB) across all income levels.

### Affordability of Medicines

The affordability of 30-day treatment of each medicine was calculated using the minimum wage for the lowest-paid unskilled government worker in each country. Thirty-day treatment of all the medicines were affordable in most of the surveyed countries except in few countries in low-income group like Tanzania (**Table 4**). For 1 month’s treatment with metformin 500 mg (twice a day for 30 days), patients would have to spend more than 5 day’s wages for the IB and 2.5 day’s wages for LPG in Tanzania. Conversely, for the same medicine, patients would have to spend ≤0.5 day’s wages in middle- and high-income countries. In case of affordability, significant difference was noted between regional groupings for IB insulin 100 IU/ml, where a significant difference was observed between low-income and high-income countries (2.37 *vs*. 0.46, *p = 0.038*) suggesting that IB insulin 100 IU/ml is more affordable in high-income compared to low-income countries.

**Table 4:** Affordability and median price-ratio comparisons for selected medicines, innovator brand, and their equivalent generic, by income level.

Medicines		Lower income		Lower-middle income		Upper-middle income		High income	
		Affordability	Price-ratio	Affordability	Price-ratio	Affordability	Price-ratio	Affordability	Price-ratio
Metformin 500 mg	IB	**2.63**	**3.15**	0.32	1.82	0.52	6.60	0.29	**3.50**
LPG		0.88	1.17	0.27	1.89	0.28	**3.52**	0.11	1.65
Metformin 850 mg XR	IB	0.06	0.38	0.33	2.19	0.25	**3.23**	0.11	**7.26**
LPG		0.52	1.18	0.26	2.12	0.13	2.06	0.10	0.70
Gliclazide 80 mg	IB	–	–	0.44	0.64	0.04	2.82	0.02	3.85
LPG		0.01	0.10	0.23	0.80	0.04	2.98	0.03	2.75
Insulin 100 IU/ml	IB	**2.37**	0.58	0.12	2.16	0.29	0.75	0.46	0.92
LPG	–	–	–	0.12	2.36	0.07	1.21	0.02	0.20

For median price-ratio, a value ≥3 in the private sector means the local’s pay more for the medicine than recommended by WHO [22, 29]. The WHO recommend that for a treatment to be affordable it should not exceed 1 day’s wages using the wage of the lowest-paid unskilled government worker. Bolded figures show price-ratio more than WHO recommended value of ≥3 in the private sector OR when figure exceeded 1 day’s wages. Bolded figures represents statistical significance = p < 0.05.

### Prices of Medicines

Overall, IBs were more expensive than LPGs but not in all cases (**Table 4**). For example, the price for IB of metformin 500 mg was less than the prices of LPG in facilities in Afghanistan, Armenia, Egypt, Georgia, and Saudi Arabia. This was also the case for insulin 100 units/ml in Jordan and Oman.

The MPRs for insulin 100 IU/ml (IB and LPG) were found to be higher in low and lower-middle income countries. For insulin 100 IU/ml, countries in low-income paid 2.37 times the IRP while countries in high-income group paid only 0.92 times the IRP to procure IB.

In the present study, it has been found that countries paid 0.10 to 7.26 times the IPRs to procure surveyed medicines. The MPR for IB metformin 500 mg was higher in upper-middle- and high-income countries than low- and lower-income countries. Countries in upper-middle and high-income paid 6.60 and 3.50 times the IRP to procure IB of metformin 500 mg, respectively. In case of LPG metformin 500 mg, MRP was highest in upper middle-income countries (countries in this group paid 3.52 times the IRP to LPG of metformin 500 mg).

The MPR for gliclazide 80 mg (IB and LPG) were found to be higher in upper middle- and high-income countries compared with low-income countries. For example, countries in lower-middle income paid 0.44 times the IRP to procure IB of gliclazide while countries in high-income group paid 3.85 times the IRP. In pairwise comparisons, statistically significant differences in median price ratios between income levels were only noted for Diamicron (gliclazide 80 mg) (p = 0.007).

**Table 5** presents foreign exchange rates and PPP adjusted median prices for 30-days treatment with IBs. When using foreign exchange rates, the median drug price of insulin 100 IU/ml was highest in the Oman ($4.82) and for the remaining six countries, the range of median prices was from $0.043 to $2.52. When using purchasing power parity to convert from local currency to US$, the pattern of prices was different. The highest PPP-adjusted price for insulin 100 IU/ml was in Bangladesh ($80.21). Both, Sri Lanka ($30.50) and India ($30.79) had similar PPP adjusted median prices for insulin. The PPP-adjusted prices for metformin 500 mg was highest in Tanzania ($4334.17) followed by Armenia ($1479.87).

**Table 5:** Median prices for 30-days treatment with diabetes medicines, by foreign exchange rates, and purchasing power parity adjustment.

Countries	Metformin 500 mg		Metformin 850 XR		Gliclazide 80 mg	Insulin 100 IU/ml	
	XR*	PPP**	XR*	PPP**	XR*	XR*	PPP**
**Afghanistan**	1.92	40.28	–	–	–	–	–
**Armenia**	7.38	1479.87	–	–	–	–	–
**Bangladesh**	4.31	137.18	4.32	137.63	2.52	2.52	80.21
**Egypt**	1.07	3.64	0.67	2.29	0.71	0.71	2.44
**Georgia**	5.76	5.64	–	–	–	–	–
**India**	0.02	0.39	2.38	42.92	1.71	1.71	30.79
**Jordan**	4.23	1.35	–	–	–	–	–
**Nepal**	0.39	13.39	0.15	5.25	0.04	0.04	1.49
**Oman**	2.79	0.45	2.16	0.34	4.82	4.82	0.77
**Pakistan**	0.83	25.71	0.73	22.79	0.88	0.88	27.18
**Qatar**	4.53	8.48	–	–	–	–	–
**Russia**	2.26	52.99	2.54	59.59	–	–	–
**Saudi Arabia**	201.60	306.43	–	–	–	–	–
**Sri Lanka**	0.44	21.88	–	–	0.61	0.61	30.50
**Tanzania**	5.93	4334.17	5.27	3852.59	–	–	–
**United Arab Emirates**	1.76	0.04	–	–	–	–	–

*XR, price in US$ when converted from local currency using foreign exchange rates.

**PPP, price in US$ when converted from local currency using purchasing power parity.

## Discussion

The present study compared the availability, prices, and affordability of four diabetes medicines across 17 countries. Medicines used to treat diabetes are listed on the WHO EML but the access to these medicines is still a worldwide concern. In the majority of lower- and middle-income countries the private sector retail pharmacies are the preferred place to buy medicines. One of the reasons for this trend is that these have a higher availability than public pharmacies, and often also more accessible in terms of geographical location (Syhakhang, 2001). Our findings are similar to previous findings on chronic conditions in low- and middle-income countries (Syhakhang, 2001; Mendis et al., 2007; Cameron et al., 2009; Wang et al., 2017). Generally, LPGs are more readily available than IBs. For instance, India and neighboring countries had more LPGs than IBs available (Kotwani, 2009; Wang et al., 2017). This is not surprising as India has a high production of generic medicines. We suggest that governments should be looking at policies to enhance the routine availability of low cost good quality generics (and in the long term striving for Universal Health Coverage) in growing areas of concern including hypertension and type 2 diabetes.

Compared to previously conducted studies not much improvement in availability has been observed. Ewen et al. (2017) performed the secondary analysis of 30 surveys undertaken in low-income and middle-income countries from 2008 to early 2015 using the WHO/HAI methodology. They reported median percentage availability (in private sector for any diabetes product) of 69.5% (IBs: 12.1% and LPGs: 65.2%) in low-income, 71.0% (IBs: 24.0%, LPGs: 66.2%) in lower- and middle-income, and 89.9% (IBs: 60.0%, LPGs: 71.7%) in upper-middle income countries (Ewen et al., 2017). The median percentage availability of LPGs of metformin (500 and 850 mg) and insulin 100 IU/ml was lower in present study compared with previous studies as shown in **Table 6**.

**Table 6:** Comparison of availability of diabetes medicines.

Medicines	Availability of LPGs (No. of studies)^a^	Current study
Metformin 500 and 850 mg cap/tab	76.7% (n = 23)	70.97%
Insulin human, soluble, isophane and 30/70, 100 IU/ml vial	30.0% (n = 9)	26.0%
Gliclazide 80 mg cap/tab	13.3% (n = 4)	46.0%

a Ewen M, Zweekhorst M, Regeer B, Laing R (2017) Baseline assessment of WHO’s target for both availability and affordability of essential medicines to treat non-communicable diseases. PLoS ONE 12(2): e0171284.

Given the low mean availability of medicines in LMICs measures must be taken to increase the availability (Babar et al., 2011; Toverud et al., 2015; Flood et al., 2017). The analysis of availability of a given medicine is important when laying the strategy to implement easy and affordable access to medicines. For example, factors contributing to poor availability of insulin such as its quantification at the national level, in-country distribution, and determination of needs at lower levels of the health system should be addressed to improve insulin availability (Beran et al., 2016). Beran *et al*., argued that very little has been done globally to address the issue of access, despite the United Nation’s political commitment (Beran et al., 2016). Previous literature from the Jiangsu province in China showed implementation of national policies to have a positive impact on the availability (Xi, 2015).

Significant differences were observed in the prices of surveyed medicines in low-, middle-, and high-income countries (using foreign exchange rates and PPP adjusted). The differences in prices of IBs obtained using foreign exchange rates and PPP adjustment were significant in low- and middle-income countries (e.g., in Armenia and Tanzania), while these differences were insignificant in high-income countries (e.g., Oman and Qatar). One of the main reasons is the difference in the value of US currency between the countries.

On the international market, the diabetes medicines are available at relatively low costs; for example, the median monthly treatment costs in 2015 were US$ 0.50 for gliclazide 80 mg, US$ 1.94 for metformin, and US$ 4.35 for regular or neutral protamine Hagedorn insulin (WHO Collaborating Centre for Drug Statistics Methodology, 2018). In this study, most of the surveyed countries had higher monthly treatment costs for metformin and gliclazide and lower monthly treatment costs for insulin than international market. One approach to improve affordability could be increasing the availability of low cost metformin and low cost biosimilar insulins which can help address the growing burden of diabetes across countries.

**Figure 1:**
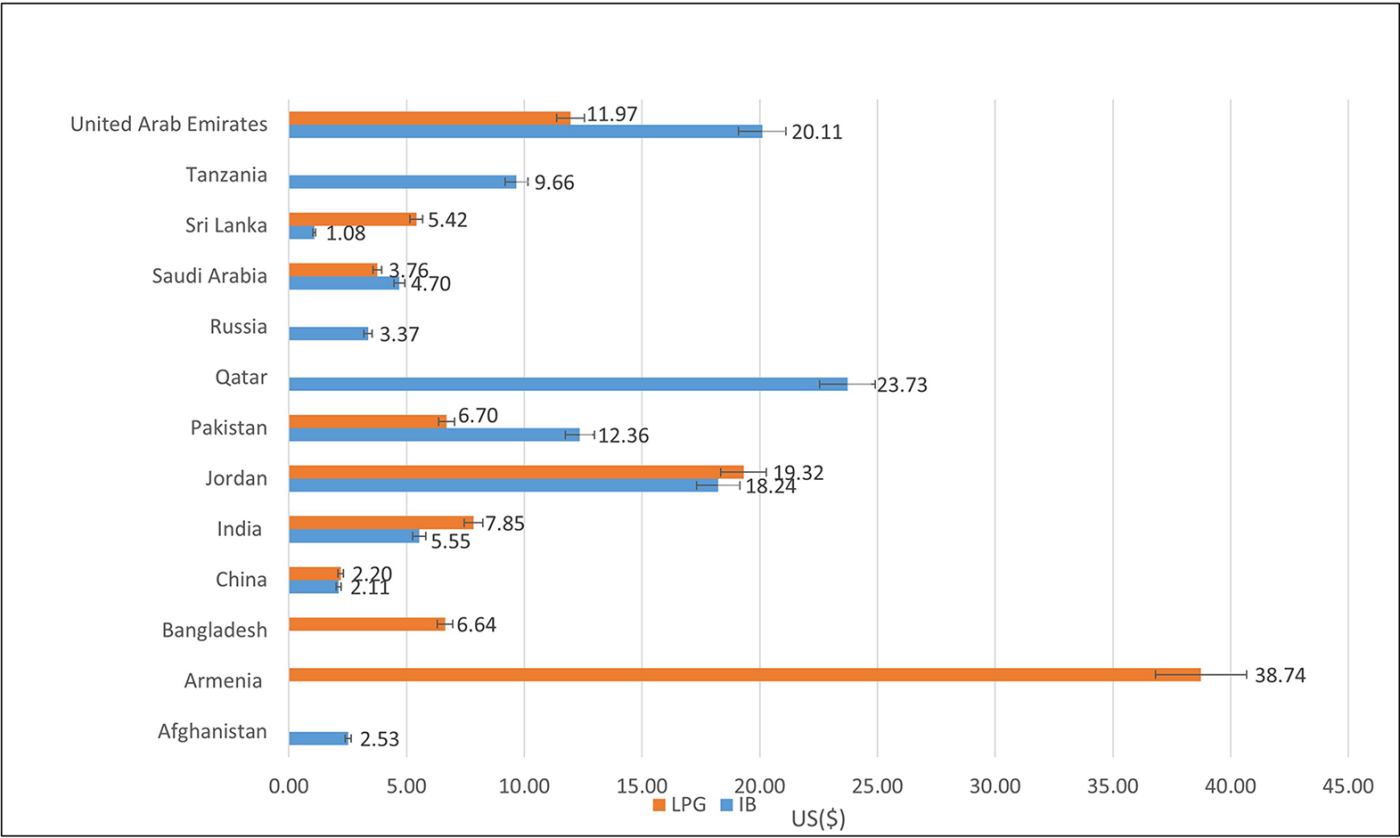
Median prices of 30-day insulin treatment (Human Regular Insulin 100 IU) prices for Innovator Brand and Lowest Prices Generic expressed in US$ across 13 countries.

The present study found that the prices of 30-day insulin 100 IU/ml treatment vary across countries (**Figure 1**). A parallel pattern of varied insulin prices is observed in previous studies (Beran et al., 2016; Liu et al., 2017). Insulin was found to be more affordable in middle- and high-income countries than low-income countries. One study in Hubei province in China found that at least four day’s wages were required to pay for 1-month treatment with insulin 100 IU/ml (Human Regular) (Liu et al., 2017). Novo Nordisk, a lead insulin manufacturer, have developed a differential pricing mechanism in order to increase affordability of insulin (Nordisk, 2017). To combat the difference in price it has been suggested that the WHO establish a drug information facility which would report on price, safety, effectiveness, and quality-assurance of essential medicines (Beran et al., 2016). The literature also suggests, substantially higher prices of new insulin analogues and diabetes medicines and their additional costs to individuals. These systems need to be assessed in terms of added clinical benefit and financial impact (Gelders et al., 2006; Beran et al., 2016).

In this study, median number of day’s wages needed to procure IBs and LPGs of four surveyed diabetes medicines were 1.69 and 0.47 in low-income; 0.30 and 0.22 in lower-middle income; and 0.27 and 0.13 in upper-middle income countries. Median number of days’ wages needed to purchase standard diabetes treatments with IBs and LPGs elsewhere were 5.3 and 1.1 in low-income, 3.8 and 1.4 in lower-middle income, 1.4 and 0.5 in upper-middle income countries (Beran et al., 2016; Ewen et al., 2017).

Tanzania is one country in this study, where we found very low median percentage availability of surveyed medicines and 1-month’s treatment with metformin 500 mg (twice a day for 30 days) costs more than 5 day’s wages for the IB and 2.5 day’s wages for LPG. This could be due to selection of EM or lack of economic evaluations when listing medicines. Mori and colleagues (Mori et al., 2014) report on how the selection of EMs in Tanzania is based on the committees of experts’ experience and discretionary judgment rather than evidence-based research. The authors found that low availability of pharmacoeconomic studies in Tanzania could be one reason for this trend as well as lack of training in evidence-based decision-making.

As reported by Ewen et al. (2017), one of the goals for Global Action Plan for the Prevention and Control of NCDs 2013–2020 (GAP) was to improve patient access to affordable medicines. They underline the importance of increased commitment and national interventions in order for the project to be successful. They also found that there was a low availability and/or affordability of essential medicines. Most often the medicines are not available in the public sector, which forces the patients to seek the private sector.

Enforcing national policies and recommendations for pro-generic prescribing could encounter the low or no availability of essential medicines. Generally, the use of generic medicines provides cheaper alternate options and lower the expenditure for patients who are paying out-of-pocket. However, generic medicines often cost more than 2 to 3 times the IRP in the public sector due to manufacturers’ selling prices, taxes, and mark-up in the supply chain (Cameron et al., 2009). Some LPGs were found to be costlier than the corresponding IB in this study.

Hassali and colleagues reviewed experiences with successful implementation of generic substitution in eight selected countries (Hassali et al., 2014). Among others they found that some generic medicines are priced more than expected or original brand medicines in some countries (Hassali et al., 2014).

It is important to understand that there are different policies implemented to promote generic medicines, for example, generic substitution in the US, generic prescribing in the UK, and mandatory generic substitution in Sweden and Finland (Hassali et al., 2014). One important factor that play a role is perceptions of healthcare professional and patients. Previous studies show that although the use of generic medicines is more affordable, but patients and healthcare professionals hold negative perceptions regarding the use of generic medicines (Shafie and Hassali, 2008; Hassali et al., 2014; Colgan et al., 2015). An understanding of their perceptions and attitudes toward the use of generic medicines would allow for a more cost-effective use of medicines (Shafie and Hassali, 2008; Colgan et al., 2015). Healthcare professionals also play a vital role in educating patients about generic medicines through counseling and educational campaigns.

Recently, Department of Health in Abu Dhabi, United Arab Emirates has authorized generic prescribing as first choice of dispensing (Abu Dhabi pharmacies told to dispense generic medicines in new regulation, 2018; Generic drugs rule will keep costs down). If the patient wants the higher priced original brand, they can buy this by paying for the difference between the lowest generic and innovator. This model is also used in high-income countries in Europe e.g., Denmark, Sweden, and Finland.

### Limitations and Recommendations for Future Studies

This study has a number of limitations. First, data were collected for few strengths of the three diabetes essential medicines only. Second, this is a point-in-time sampling study and data were only collected from at least two private retail pharmacy sites per country (range: 2 –4), therefore the results are less generalizable than if multiple sites were sampled within countries. Third, as we have applied purposive sampling, countries with active collaborations with authors were more likely to be sampled than those who did not, and therefore might have introduced selection bias. It would have been better if more countries from sub-Saharan Africa, South America had taken part with an appreciable and growing prevalence of diabetes (principally T2DM).

Fourth, the study did not capture the data about level of patient co-payment for oral anti-diabetic medicines or educational input prescribers receive to help make sense of the findings as there can be high prices—but this is not an issue in LMICs with universal access since in these countries medicines are provided free of charge in government facilities. The real issues are adherence to medicines once prescribed and ensuring their availability in primary healthcare centers (as this can necessitate purchasing directly from community pharmacies). Also, we did not explore the concerns with generics in these countries including issues of quality.

Fifth, a diverse definition of “availability” used across studies. The WHO/HAI standardized method calls for using specific strength and dosage form as indicated in collection forms. This methodology is not always followed, some studies define a medicine to be available regardless of the available IB/generic and in some cases also strength of the medicine (Dabare et al., 2014; Hassali et al., 2014). A more rigid approach to the definition of availability would allow to track trends over time and hence identify which essential medicine are available at which strength and price.

Sixth, the definition of “selling price” varies across studies. Many studies including current study do not take mark-ups, taxes, and other distribution costs under consideration when calculating and comparing prices. For future studies it would be essential to include a measure of the weakness in the supply system to identify action required to increase the availability and affordability of EM.

Finally, there are limitations that are inherent from WHO/HAI’s methodology. For example, this study has used the wage of lowest-paid unskilled government worker to calculate affordability. There may be individuals who earn less and hence data may not be a true reflection of the reality (World Health Organization, 2004; Cameron et al., 2009). Incorporate other living fixed costs such as food, electricity, water, home rent when measuring affordability. Leading medicine and pharmaceutical pricing researchers have proposed an alternative metric to express affordability, but this is not yet readily used.

## Conclusions

Overall the availability of four essential diabetes medicines was found to be sub-optimal in the primary care pharmacy settings. Across the 17 countries, some facilities did not have any branded products available. Although the availability of the generic form of insulin was found to be poor, we also found a lower monthly treatment cost compared to the international market. The four medicines have an acceptable median price-ratio and affordability in most of the surveyed countries. There is a need to move from *ad hoc* study culture to studies that follow standardized methodologies to support evidence-based decision-making.

## Data Availability Statement

The datasets for this manuscript are not publicly available. The datasets obtained as a part of this will be available on request. Requests to access the datasets should be directed to z.babar@hud.ac.uk.

## Author Contributions

ZB conceptualized the project with SH, IT, and AA. FE-D contributed with data collection. SH contributed to data analysis, interpretation, and manuscript development. ZB and SR contributed with data collection, data entry, data analysis, and manuscript development. The final version was approved by all authors.

## Funding

This project is funded by University of Huddersfield (GCRF Sandpit Project 2017/18).

## Conflict of Interest

The authors declare that the research was conducted in the absence of any commercial or financial relationships that could be construed as a potential conflict of interest.

## References

[B1] Abu Dhabi pharmacies told to dispense generic medicines in new regulation (2018). [cited 2019 February 3rd]; Available from: https://gulfnews.com/uae/health/abu-dhabi-pharmacies-told-to-dispense-generic-medicines-in-new-regulation-1.2260407.

[B2] Babar Z. U. Ibrahim M. I. Singh H. Bukhari N. I. Creese A. (2007). Evaluating drug prices, availability, affordability, and price components: implications for access to drugs in Malaysia. PloS Med. 4 (3), e82. 10.1371/journal.pmed.0040082 17388660 PMC1831730

[B3] Babar Z. U. Grover P. Stewart M. Hogg J. Seo H. G. Rew A. (2011). Evaluating pharmacists' views, knowledge, and perception regarding generic medicines in New Zealand. Res. Soc. Adm. Pharm. 7 (3), 294–305. 10.1016/j.sapharm.2010.06.004 21272551

[B4] Babar Z. U. Lessing C. Mace C. Bissell K. (2013). The availability, pricing and affordability of three essential asthma medicines in 52 low- and middle-income countries. Pharmacoeconomics 31 (11), 1063–1082. 10.1007/s40273-013-0095-9 24127259

[B5] Beran D. Ewen M. Laing R. (2016). Constraints and challenges in access to insulin: a global perspective. Lancet Diabetes Endocrinol. 4 (3), 275–285. 10.1016/S2213-8587(15)00521-5 26857998

[B6] Beran D. Ewen M. Lipska K. Hirsch I. B. Yudkin J. S. (2018). Availability and affordability of essential medicines: implications for global diabetes treatment. Curr. Diabetes Rep. 18 (8), 48. 10.1007/s11892-018-1019-z 29907884

[B7] Cameron A. Ewen M. Ross-Degnan D. Ball D. Laing R. (2009). Medicine prices, availability, and affordability in 36 developing and middle-income countries: a secondary analysis. Lancet 373 (9659), 240–249. 10.1016/S0140-6736(08)61762-6 19042012

[B8] Colgan S. Faasse K. Martin L. R. Stephens M. H. Grey A. Petrie K. J. (2015). Perceptions of generic medication in the general population, doctors and pharmacists: a systematic review. BMJ Open 5 (12), e008915. 10.1136/bmjopen-2015-008915 PMC4679988 26671954

[B9] Dabare P. R. Wanigatunge C. A. Beneragama B. H. (2014). A national survey on availability, price and affordability of selected essential medicines for non-communicable diseases in Sri Lanka. BMC Public Health 14, 817. 10.1186/1471-2458-14-817 25103467 PMC4138405

[B10] Ewen M. Zweekhorst M. Regeer B. Laing R. (2017). Baseline assessment of WHO's target for both availability and affordability of essential medicines to treat non-communicable diseases. PloS One 12 (2), e0171284. 10.1371/journal.pone.0171284 28170413 PMC5295694

[B11] Flood D. Mathieu I. Garcia P. Rohloff P. (2017). Perceptions and utilization of generic medicines in Guatemala: a mixed-methods study with physicians and pharmacy staff. BMC Health Serv. Res. 17 (1), 27. 10.1186/s12913-017-1991-z 28086866 PMC5234139

[B12] Forex exchange rates Available from: https://www.dailyfx.com/charts?ref=SubNav Accessed on 24/09/2018 at 16.40.

[B13] Gelders S. E. Noguchi N. Laing R. (2006). Price, availability and affordability: an international comparison of chronic disease medicines [WHO-EM/EDB/068/E]. [cited 2018 June 21st]; Available from: http://www.who.int/iris/handle/10665/116493.

[B14] Generic drugs rule will keep costs down [cited 2019 February 3rd]; Available from: https://www.khaleejtimes.com/news/uae-health/generic-drugs-rule-will-keep-costs-down.

[B15] Gustafsson L. L. Wettermark B. Godman B. Andersén-Karlsson E Bergman U Hasselström J(2011). The 'wise list'- a comprehensive concept to select, communicate and achieve adherence to recommendations of essential drugs in ambulatory care in Stockholm. Basic Clin. Pharmacol. Toxicol. 108, 224–233. 10.1111/j.1742-7843.2011.00682.x 21414143

[B16] HAI (2008). Measuring medicine prices, availability, affordability and price components. [cited 2018 July 17th]; Available from: http://www.haiweb.org/medicineprices/manual/documents.html.

[B17] Hassali M. A. Alrasheedy A. A. McLachlan A. Nguyen T. A. Al-Tamimi S. K. Ibrahim M. I. (2014). The experiences of implementing generic medicine policy in eight countries: A review and recommendations for a successful promotion of generic medicine use. Saudi Pharm. J. 22 (6), 491–503. 10.1016/j.jsps.2013.12.017 25561861 PMC4281627

[B18] IDF (2018). International Diabetes Federation (IDF) diabetes Atlas, [cited 2018, 25th March]; Available from: http://www.diabetesatlas.org/. 35914061

[B19] Kotwani A. (2009). Availability, price and affordability of asthma medicines in five Indian states. Int. J. Tuberc. Lung Dis. 13 (5), 574–579. 19383189

[B20] Liu C. Zhang X. Liu C. Ewen M. Zhang Z. Liu G. (2017). Insulin prices, availability and affordability: a cross-sectional survey of pharmacies in Hubei Province, China. BMC Health Serv. Res. 17 (1), 597. 10.1186/s12913-017-2553-0 28836974 PMC5571633

[B21] Mathers C. D. Loncar D. (2006). Projections of global mortality and burden of disease from 2002 to 2030. PloS Med. 3 (11), e442. 10.1371/journal.pmed.0030442 17132052 PMC1664601

[B22] McEwen L. N. Casagrande S. S. Kuo S. Herman W. H. (2017). Why are diabetes medications so expensive and what can be done to control their cost? Curr. Diabetes Rep. 17 (9), 71. 10.1007/s11892-017-0893-0 28741264

[B23] Mendis S. Fukino K. Cameron A. Laing R. Jr Filipe A. Khatib O., (2007). The availability and affordability of selected essential medicines for chronic diseases in six low- and middle-income countries. Bull. World Health Organ. 85 (4), 279. 10.2471/BLT.06.033647 17546309 PMC2636320

[B24] Mori A. T. Robberstad B. (2012). Pharmacoeconomics and its implication on priority-setting for essential medicines in Tanzania: a systematic review. BMC Med. Inform. Decis. Mak. 12, 110. 10.1186/1472-6947-12-110 23016739 PMC3472274

[B25] Mori A. T. Kaale E. A. Ngalesoni F. Norheim O. F. Robberstad B. (2014). The role of evidence in the decision-making process of selecting essential medicines in developing countries: the case of Tanzania. PloS One 9 (1), e84824. 10.1371/journal.pone.0084824 24416293 PMC3885598

[B26] MSH (2015). The International Medical Products Price Guide [cited 2018 March 15th]; Available from: http://mshpriceguide.org/en/home/.

[B27] Nordisk N. (2017). Novo Nordisk Annual Report 2017., Novo Nordisk.

[B28] Opinions of the Communist Party of China Central Committee and the State Council on Deepening the Health Care System Reform (2009). http://www.china.org.cn/government/scio-press-conferences/2009-04/09/content_17575378.htm

[B29] Shafie A. A. Hassali M. A. (2008). Price comparison between innovator and generic medicines sold by community pharmacies in the state of penang, Malaysia. J. Generic Med. 6 (1), 35–42. 10.1057/jgm.2008.25

[B30] Shi L. Hodges M. Drummond M. Ahn J. Li S. C. Hu S., (2010). Good research practices for measuring drug costs in cost-effectiveness analyses: an international perspective: the ISPOR Drug Cost Task Force report-Part VI. Value Health 13 (1), 28–33. 10.1111/j.1524-4733.2009.00662.x 19883403

[B31] Syhakhang L. Stenson B. Wahlström R. Tomson G. (2001). The quality of public and private pharmacy practices. A cross sectional study in the Savannakhet province, Lao PDR. Eur. J. Clin. Pharmacol. 57 (3), 221–227. 10.1007/s002280100295 11497337

[B32] The World Bank (2018a). Low income. [cited 2018 April 20th]; Available from: https://data.worldbank.org/income-level/low-income.

[B33] The World Bank (2018b). Lower middle income. [cited 2018 April 20th]; Available from: https://data.worldbank.org/income-level/lower-middle-income.

[B34] The World Bank (2018c). Upper middle income. [cited 2018 April 20th]; Available from: https://data.worldbank.org/income-level/upper-middle-income.

[B35] The World Bank (2018d). High income. [cited 2018 April 20th]; Available from: https://data.worldbank.org/income-level/high-income.

[B36] Toverud E. L. Hartmann K. Hakonsen H. (2015). A systematic review of physicians' and pharmacists' perspectives on generic drug use: What are the Global Challenges?. Appl. Health Econ. Health Policy 13 (1), S35–S45. 10.1007/s40258-014-0145-2 25963230 PMC4519583

[B37] van Mourik M. S. Cameron A. Ewen M. Laing R. O. (2010). Availability, price and affordability of cardiovascular medicines: A comparison across 36 countries using WHO/HAI data. BMC Cardiovasc. Disord. 10 (1), 25. 10.1186/1471-2261-10-25 20534118 PMC2898673

[B38] Wang H. Sun Q. Vitry A. Nguyen T. A. (2017). Availability, price, and affordability of selected essential medicines for chronic diseases in 11 countries of the asia pacific region: a secondary analysis. Asia Pac. J. Public Health 29 (4), 268–277. 10.1177/1010539517700472 28397532

[B39] WHO Collaborating Centre for Drug Statistics Methodology (2018). Drugs used in diabetes. Oslo: Norwegian Institute of Public Health http://www.whocc.no/atc_ddd_index/?code=A10A Accessed 1 February 2019.

[B40] WHO (2018a). . Medicines Pricing and Financing, [cited 2018 July 13th]; Available from: http://www.who.int/medicines/areas/access/en/.

[B41] WHO (2018a). Essential medicines, [cited 2018 July 16th]; Available from: http://www.who.int/medicines/services/essmedicines_def/en/.

[B42] WHO (2018c). . Collaborating Centre for Drug Statistics Methodology, Drugs used in diabetes[cited 2018 1st October]; Available from: http://www.whocc.no/atc_ddd_index/?code=A10A.

[B43] World economic outloook Available from: https://www.imf.org/external/datamapper/PPPEX@WEO/OEMDC/ADVEC/WEOWORLD/AFG accessed on 24/09/2018 at 1630.

[B44] World Health Organization (2004). WHO medicines strategy. Geneva, Switzerland: Countries at the core 2004-2007, Available from: http://apps.who.int/medicinedocs/pdf/s5416e/s5416e.pdf [16/10/2019].

[B45] Xi X. Li W. Li J. Zhu X. Fu C. Wei X. (2015). A survey of the availability, prices and affordability of essential medicines in Jiangsu Province, China. BMC Health Serv. Res. 15 (1), 345. 10.1186/s12913-015-1008-8 26310243 PMC4549946

